# Efficient Sensing of Avian Influenza Viruses by Porcine Plasmacytoid Dendritic Cells

**DOI:** 10.3390/v3040312

**Published:** 2011-03-30

**Authors:** Michael Bel, Manuela Ocaña-Macchi, Matthias Liniger, Kenneth C. McCullough, Mikhail Matrosovich, Artur Summerfield

**Affiliations:** 1 Institute of Virology and Immunoprophylaxis, Sensemattstrasse 293, CH-3147 Mittelhäusern, Switzerland; E-Mails: michael.bel@hcuge.ch (M.B.); manuela.ocana@ivi.admin.ch (M.O.-M.); matthias.liniger@ivi.admin.ch (M.L.); ken.mccullough@ivi.admin.ch (K.C.M.); 2 Institute of Virology, Philipps University, Hans-Meerwein-Str. 2, 35043 Marburg, Germany; E-Mail: m.matrosovich@gmail.com (M.M.)

**Keywords:** plasmacytoid dendritic cells, influenza A virus, interferon, cytokine storm

## Abstract

H5N1 influenza A virus (IAV) infections in human remain rare events but have been associated with severe disease and a higher mortality rate compared to infections with seasonal strains. An excessive release of pro-inflammatory cytokine together with a greater virus dissemination potential have been proposed to explain the high virulence observed in human and other mammalian and avian species. Among the cells involved in the cytokine storm, plasmacytoid dendritic cells (pDC) could play an important role considering their unique capacity to secrete massive amounts of type I interferon (IFN). Considering the role of IFN as a major component of antiviral responses as well as in priming inflammatory responses, we aimed to characterize the induction of IFN-α release upon infection with IAV originating from various avian and mammalian species in a comparative way. In our porcine pDC model, we showed that the viral components triggering IFN responses related to the ability to hemagglutinate, although virosomes devoid of viral RNA were non-stimulatory. Heat-treatment at 65 °C but not chemical inactivation destroyed the ability of IAV to stimulate pDC. All IAV tested induced IFN-α but at different levels and showed different dose-dependencies. H5 and H7 subtypes, in particular H5N1, stimulated pDC at lower doses when compared to mammalian IAV. At high viral doses, IFN-α levels reached by some mammalian IAV surpassed those induced by avian isolates. Although sialic acid-dependent entry was demonstrated, the α-2,3 or α-2,6 binding specificity alone did not explain the differences observed. Furthermore, we were unable to identify a clear role of the hemagglutinin, as the IFN-α doses-response profiles did not clearly differ when viruses with all genes of identical avian origin but different HA were compared. This was found with IAV bearing an HA derived from either a low, a high pathogenic H5N1, or a human H3. Stimulation of pDC was associated with pDC depletion within the cultures. Taken together and considering the efficient sensing of H5N1 at low dose, pDC on one side may play a role in the cytokine storm observed during severe disease, on the other hand could participate in early antiviral responses limiting virus replication.

## 1. Introduction

The first human infection with the H5N1 high pathogenic avian influenza virus (HPAIV) occurred in 1997, and since then many other human cases were confirmed coinciding with outbreaks in poultry mainly in Asia. Although inefficient in human-to-human transmission, this newly emerged H5N1 HPAIV caused high mortality above 50% in human. Infected individuals developed a severe pneumonia with greater systemic virus dissemination [1], contrasting with the respiratory tract-restricted tropism mostly found after infection with human influenza A virus (IAV). The tissue damage found in the lung as well as in other organs is believed to be due mainly to a dysregulation of innate immune responses leading to a “cytokine storm” characterized by the presence of elevated levels of pro-inflammatory cytokines and interferons (IFN) in several tissues such in the respiratory tract and the blood [2]. Also, *in vitro* infection of primary respiratory epithelial cells showed abundant transcription and expression of interleukin-(IL)6, and certain chemokines [3]. Moreover, macrophages infected with human H5N1 isolates were found to express elevated levels of IFN-α, IL-1β, tumor-necrosis-factor (TNF)-α and chemokines *in vitro* [4]. Cells of the innate immunity are the first to be recruited to the site of infection and therefore contribute, together with lung epithelial cells, to the cytokine and chemokine release to orchestrate immune responses.

Among these innate immune mediators, type I IFN is a key component of anti-viral immunity by limiting influenza virus replication and inducing adaptive immune responses [5]. Among innate immune cells, plasmacytoid dendritic cells (pDC), a subset of the DC family, are highly specialized in virus sensing. pDC possess the unique capacity to produce and secrete 100–1000 times more type I IFN than any other cell type in response to virus stimulation [6]. Furthermore, pDC were shown to be the major contributors of IFN-α in the lungs of mice upon IAV infection [7,8]. Considering the distinct features of pDC and the crucial role of IFN in fighting virus infections, we reasoned that these cells could play an important role in promoting inflammation and thus being partly responsible for the cytokine responses and the disease development. Consequently, we analyzed the interactions of different IAV isolated from avian species, human and swine with pDC obtained from pigs, a natural host of IAV. Our results demonstrate that porcine pDC can produce high levels of IFN-α in response to all the strains tested, with subtype-specific differences in a virus-dose dependent manner. We also employed IAV generated by reverse genetics to determine the contribution of the hemagglutinin (HA) to the different responses observed between avian IAV (AIV) and mammalian IAV.

## 2. Results

### 2.1. General Characteristics of pDC Responses Induced by AIV

**Figure 1.**
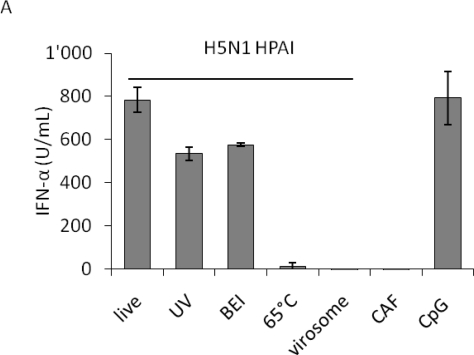

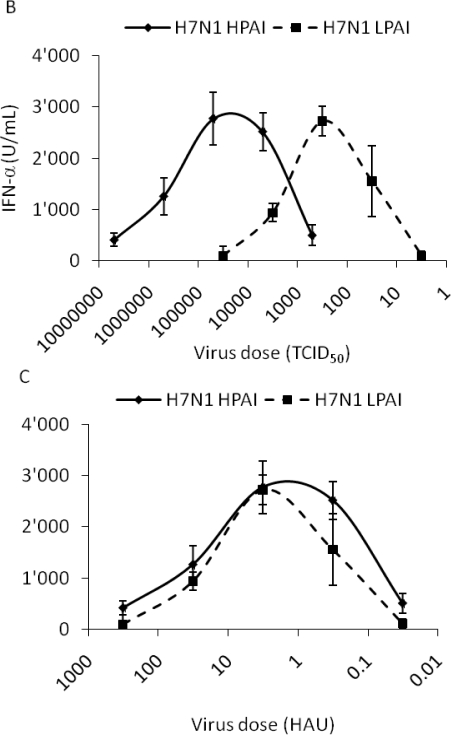
IFN-α responses of plasmacytoid dendritic cells (pDC): impact of virus inactivation, as well as virus-dose dependency using either HAU or TCID_50_ to quantify influenza A virus (IAV). Porcine pDC were enriched from fresh blood, plated and stimulated. IFN-α in the supernatant was quantified by ELISA after 24 h. (**A**) pDC were stimulated with live, UV-, 2-bromoethylamine hydrobromide (BEI)- or 65 °C heat-inactivated H5N1 virus (H5N1 TT05, see Table 1) or with H5N1-derived virosomes at a dose of 40 HAU. Allantoic fluid (CAF) or CpG (10 μg) were used as controls. Error bars represent standard deviations of triplicate ELISA samples. (**B** and **C**) pDC were stimulated with a pair of HP- and LPAIV H7N1 (H7N1 TI99 and OI00, see Table 1) at various doses. Error bars represent standard deviations of triplicate samples.

We first analyzed the capacity of porcine pDC to respond to live or inactivated H5N1 viruses to set the optimal conditions for the multiple strain comparison. High IFN-α levels were detected upon live, chemically-inactivated or UV-irradiated virus stimulation, in a similar range than with the positive control CpG. In contrast, heat-treated virus failed to induce a response. The observation that chemical and UV-light inactivation did not abolish IFN-α release indicated that non-infectious particles are also stimulatory for pDC. The latter treatments, in contrast to the heat-treatment at 65 °C, did not abolish hemagglutination, suggesting that the integrity of the HA and its binding function are necessary to induce IFN-α responses in pDC. On the other hand, H5N1 virosomes did not activate pDC indicating that RNA molecules are required to trigger IFN-α response (Figure 1A). Interestingly, in the presence of fetal bovine serum (FBS) added at 1 h post infection (p.i.), a reduction in IFN-α responses was observed at low virus doses (data not shown). Consequently, further experiments were performed in serum-free conditions.

When comparing the dose-response curve of a closely related pair of H7N1 high and low pathogenic avian influenza virus (LPAIV), we observed that the same IFN-α responses were obtained with the LPAIV employed with around 100-times lower infectious doses determined as TCID_50_ (Figure 1B). However, when plotted against hemagglutination units (HAU) both viruses elicited a similar dose-dependent response (Figure 1C). While the low pathogenic LPAIV H7N1 virus only had a four-fold lower HAU titer, its infectious titer was around 1000-times lower (Table 1). This observation and the almost overlapping curves when HAU were employed to dose the viruses (Figure 1C), indicated that HAU dosage rather than TCID_50_ is more appropriate to compare various IAV isolates on pDC. This was also supported by the observation that inactivated virus was still potent at activating pDC (Figure 1A).

### 2.2. Interferon Response of Avian and Mammalian Strains

**Figure 2.**
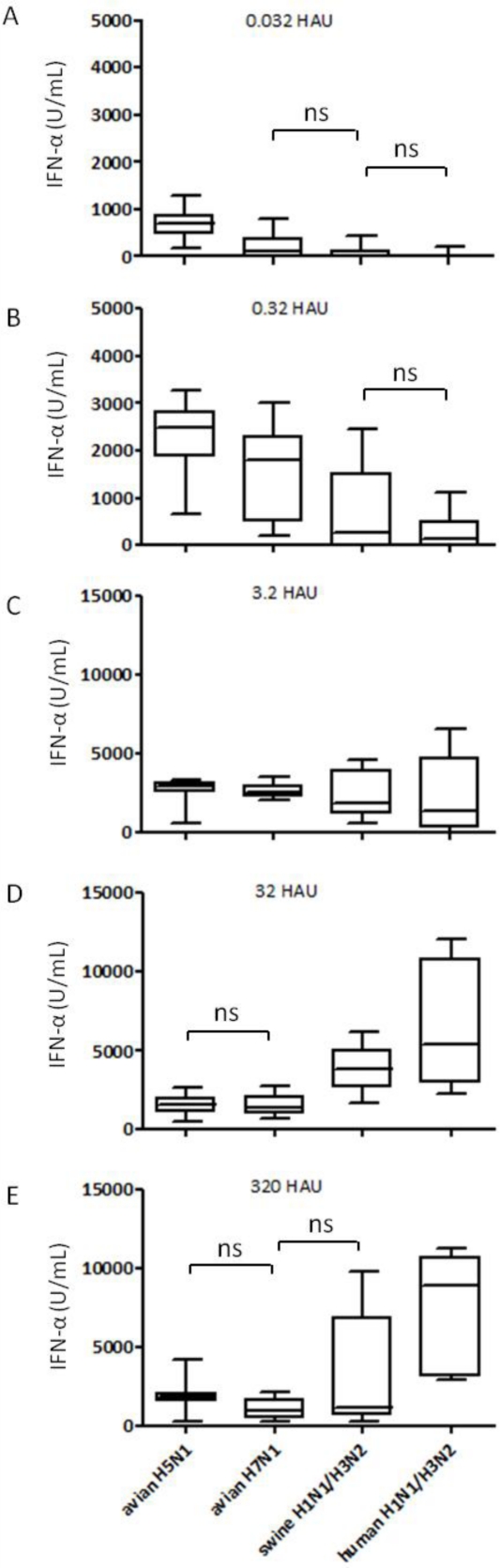
Comparative analysis of the interferogenic profiles of avian, human and swine IAV. Porcine pDC were stimulated with six different avian (H5 and H7), three human and three swine IAV (all listed in Table 1), and IFN-α in the supernatants was quantified by ELISA after 24 h. (**A**, **B**) Box plots analysis for triplicate cultures stimulated with low HAU dose of 0.032 or 0.32 (y-axis maximum 5000). (**C**, **D**, **E**) Box plots for moderate-to-high virus doses of 3.2, 32 and 320 HAU (y-axis maximum 15000). Each box was calculated from data pooled for the indicated groups (three different viruses per group, each tested in triplicate wells). Box plots analysis display data as the median and 25th and 75th percentiles and error bars the 95% confidence interval. Statistics and p values were calculated using GraphPadPrism5 Software [9]. One way repeated analysis of variance (ANOVA) was used and Bonferroni’s multiple comparisons in between the groups. Within one graph all groups are statistically different except those labeled with ns (not significant).

To compare the induction of IFN-α responses by different IAV strains we selected a few strains isolated from avian species, humans or pigs, all listed in Table 1. The six avian IAV from the H5 and H7 group, representing LP and HP strains, and the six mammalian IAV of human and porcine origin were simultaneously compared for IFN-α induction at various doses over four logs of dilutions. Our results demonstrate that pDC can sense and respond to all the strains tested although the magnitude of IFN-α responses differed in the different groups at different doses. The avian H5 and H7 viruses typically induced moderate levels of IFN-α but required lower virus doses when compared to mammalian viruses (Figure 2A, B). With these viruses, maximal IFN-α values were reached at around 3.2 HAU per well (Figure 2C), while with higher virus doses a decrease was found (Figure 2D, E). Other LPAIV isolated from water fowl with different HA-NA subtypes including H1N1, H9N2, H10N1 and H10N7 were also tested and induced similar responses at the same doses (data not shown). In contrast, the mammalian viruses tested, although more heterogenous in their stimulatory profile than their avian counterparts, induced no or low IFN-α at doses below one HAU (Figure 2A, B). In general, these viruses induced the highest IFN-α responses at the higher doses (Figure 2D, E). Statistical analyses demonstrated that at the two low doses (0.032 and 0.32 HAU) all groups differed between each other (p < 0.05) except the two mammalian groups, which almost induced no IFN-α. At 3.2 HAU avian viruses induced slightly higher responses than their mammalian counterparts although no statistical difference was found between the four groups (Figure 2C). At the higher doses of 32 and 320 HAU, a significant difference was again calculated between the avian and the mammalian groups. Furthermore, the swine and the human isolates differed in a statistically significant way.

### 2.3. IFN-α Responses are not Correlated to Viral RNA Content

As mentioned above, the presence of viral RNA is likely necessary to trigger an IFN-α response in pDC and their concentration might influence the magnitude of IFN-α and thus partly explain the different interferogenic profiles obtained. Consequently, the viral RNA content of each virus stock was quantified by real-time (RT)-PCR. The results shown in Table 1 indicate that the relative amount of viral RNA shown as CT value, although often higher with the avian isolates, did not strictly relate to the ability of some viruses to stimulate pDC at doses below 1 HAU per well.

### 2.4. Relation between pDC Percentage and IFN-α

**Figure 3.**
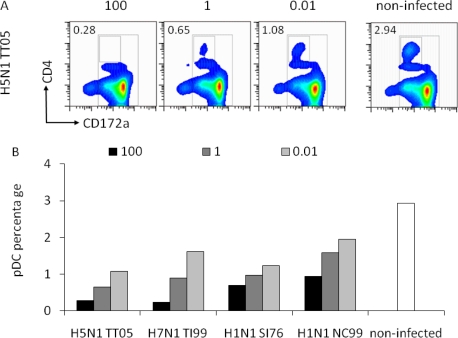

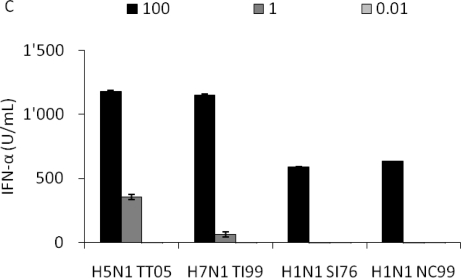
Relation between virus dose, pDC percentage and IFN-α response. Porcine pDC were stimulated with H5N1 TT05, H7N1 TI99, H1N1 SI76 and H1N1 NC99 (listed in Table 1) at 100, 1 or 0.01 HAU per well or left non-infected. (**A**) CD4/CD172a plots are shown for H5N1 TT05 infection at the tested doses and for non-infected cells to illustrate the gate for pDC definition. Numbers in the plots indicate the pDC percentage present in the small square. (**B**) pDC percentage after stimulation with different virus doses. (**C**) IFN-α secretion in the supernatant in function of the virus dose.

To further characterize the interaction between pDC and IAV, we compared the pDC percentage and the IFN-α release after infection with the avian H5N1 TT05H, H7N1 TI99L, human H1N1 NC99 and swine H3N2 SI76 at different HAU doses. Porcine pDC were identified as CD172a^low^CD4^high^ cells as exemplified for TT05H at three different HAU doses (Figure 3A). After 16 h of culture, we observed a decrease in the pDC percentages with increasing virus dose with all four selected viruses (Figure 3B). The 100 HAU dose resulted in a statistically significant lower pDC percentage that the 0.01 dose (p = 0.01). Interestingly, it appeared that the percentage of remaining pDC was inversely correlated to the amount of IFN-α released (r^2^ = 0.48).

### 2.5. Infection of pDC and Monocytic Cells

In order to determine the ability of the different IAV to infect pDC, we analyzed the viral nucleoprotein (NP) expression at 6 h p.i. in pDC and the whole CD172a^+^ cell population, which is composed of monocytes and blood DC [10]. These were gated as shown in Figure 3A and analyzed for NP expression.

**Figure 4.**
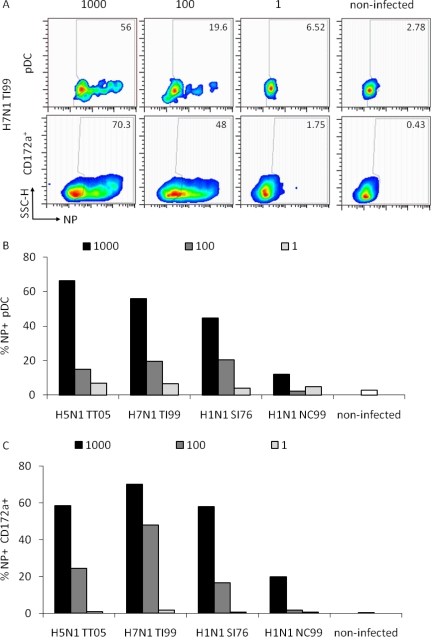
Relation between virus dose and cell infection. Porcine pDC were plated in 12-well plates and infected as in Figure 3 at 1000, 100 and 1 HAU dose or left non-infected at 37 °C. Cells were harvested, fixed and labeled for NP expression after 6 h. Cells were gated as exemplified in Figure 3 to differentiate the pDC and entire CD172a^+^ monocytic/DC population. (**A**) Side scatter/NP pseudo-color dot plots of pDC (upper panels) or the entire CD172a^+^ cells (lower panels) are shown for H7N1 TI99 infection at the three tested doses as well as for non-infected cells. Numbers in the plots indicate the percentage of NP positive cells for both populations. (**B**) Percentage of NP positive pDC in function of the virus dose. (**C**) Percentage of NP positive CD172a^+^ cells in dependence of the virus dose.

Representative data for H7N1 TI99 at three different HAU doses are shown in Figure 4A. A dose-dependent NP expression was observed for all viruses tested with a highest rate of NP^+^ pDC at the high dose of 1000 HAU/well. For pDC infection, a statistical analysis of the three doses indicated a significant difference between the 1000 and the 100 HAU dose (p = 0.05) but not between the 100 and 1 dose (p = 0.1). For the monocytes/blood DC population only the differences between the 1000 and 1 HAU dose were statistically significant (p = 0.029). We concluded that the detection of NP requires a high dose of virus. Furthermore, NP expression did not absolutely correlate to the IFN-α responses induced, since with the avian viruses HAU doses above 100 resulted in reduced IFN-α responses (Figure 1C). Furthermore, the infection rate of CD172a^+^ cells, representing mainly monocytes, was similar to that of pDC indicating no favored tropism of IAV for monocytes or pDC.

### 2.6. Sialic-Acid Dependant Entry of IAV in pDC

Sialic acid (SA) residues on the host cell membrane represent the major target for the HA of IAV, and the first step in the infection of epithelial cells. Since little information on IAV receptors of innate immune cells was available, we treated enriched pDC with neuraminidase (NA) to remove the SA residues and subsequently infected the cells with the same four viruses as in Figures 3 and 4. As a sensitive and relevant readout for infection we measured IFN-α production.

**Figure 5.**
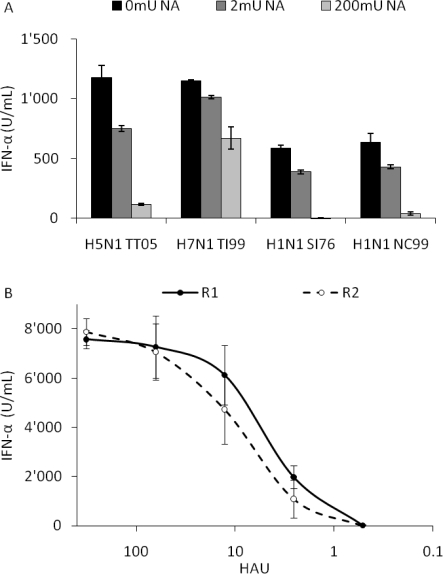
Role of sialic acid (SA) residues for IFN-α responses by pDC. (**A**) Porcine pDC were treated with 0, 2 or 200 mU of NA for 1 h, infected at a 100 HAU dose and supernatants harvested after 24 h for IFN-α quantification. (**B**) pDC were stimulated at a various HAU doses with the SA specific virus pair R1 and R2, which possess mammalian α-2,6 SA or avian-like α-2,3 SA receptor specificity, respectively. IFN-α was quantified in the supernatant by ELISA. Error bars represent the standard deviation of triplicate wells.

With increasing doses of NA the IFN-α response induced by all four viruses was reduced indicating a SA-dependent entry of IAV in pDC. With 200 mU NA, IFN-α secretions were reduced by over 90% with H5N1 TT05, swine H1N1 SI76 and human H1N1 NC99, while H7N1 TI99 was only reduced by 45% suggesting a binding also to an NA resistant receptor by this virus (Figure 5A). Interestingly, with the related HP H7N1 variant NA treatment with 200 mU also only reduced INF-α responses by 50% (data not shown). In addition the results obtained with the H7N1 TI99 virus, which still induces high levels of IFN-α at 200 mU NA, indicated that the low levels or lack of IFN-α found with the other viruses is not resulting from a possible toxicity of the NA for pDC.

### 2.7. Reverse Genetic Viruses and Interferogenic Profile

To further assess the role of HA in the different IFN-α response profiles, we employed the R1/R2 virus pairs generated by reverse genetics and differing only in two amino acids in the HA (L226Q and S228G). While R1 preferentially targeted cells bearing α-2,6 sialic acid residues and possessed the same binding specificity as the original H3N2 HK68 virus, the mutated R2 showed a favored tropism for cells with typical avian α-2,3 SA residues [11]. When this virus pair was used to infect pDC, a similar interferogenic profile was obtained indicating no role for α-2,3 *versus* α-2,6-linked SA residues in virus sensing (Figure 5B).

**Figure 6.**
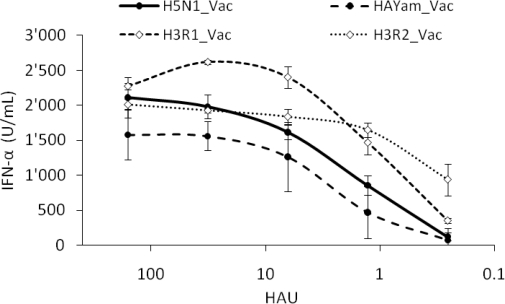
Interferogenic profile of various reverse genetic viruses. Porcine pDC were infected with various doses of the following reverse genetic virus pairs (listed in Table 2): H5N1_Vac and HAYam_Vac that carry the H5 of an LP or an HP AIV (black dots), H3R1_Vac and H3R2_Vac that bear the HA of R1 and R2 used in Figure 5, but an avian H5N1-Vac backbone (white diamonds). IFN-α in the supernatant was quantified by ELISA. Error bars represent the standard deviation of triplicate wells.

We also studied the effect of the remaining genes and created two H3N1 virus pairs with the HA from R1 or R2 and the remaining genes from the avian H5N1 vaccine strain (Vac, Table 2). When this virus pair was used to infect pDC, similar interferogenic profiles were obtained confirming not only the absence of a role of α-2,3 *versus* α-2,6-linked SA in virus sensing, but also no dominant role for other IAV genes (Figure 6).

Finally we created a third virus pair to study the potential contribution of an HA from a HPAIV. To this end, we exchanged the HA of the LP H5N1_Vac with the HA of the HP H5N1 A/Yamaguchi/7/04. Again, the two H5N1 virus pairs behaved similarly indicating no dominant role of the HA in IFN-α responses in pDC.

## 3. Experimental Section

### 3.1. Viruses and Cell Lines

All IAV (listed with defined abbreviations in Table 1) were propagated in 10–12 days old specific pathogen free embryonated chicken eggs following the WHO Manual on Animal Influenza Diagnosis and Surveillance instructions with incubation at 37 °C between 24 and 72 h depending on the pathogenicity of avian isolates. HPAIV A/Turkey/Turkey/05, A/Cygnus/Italy/742/06, A/Mallard/Italy/835/06 (H5N1), A/Turkey/Italy/4580/98, A/Ostrich/Italy/2332/00 as well as LPAIV A/Turkey/Italy/3675/99 (H7N1) were kindly provided by Dr. W. Dundon, (IZSV Istituto Zooprofilattico Sperimentale delle Venezie, Venice, Italy), human isolates A/New Caledonia/20/99 (H1N1) A/Wisconsin/67/05 (H3N2) were kindly given by Drs. W. Wunderli and Y. Thomas (National Influenza Reference Center, Geneva University Hospital, Switzerland), swine IAV A/Swine/Belgium/1/98 and A/Swine/Flanders/1/98 (H1N1) were kindly received from Dr. K. Van Reeth (Laboratory of Virology, Faculty of Veterinary Medicine, Ghent University, Belgium) and A/Hong Kong/8/68 (H3N2) and A/Swine/Iowa/1976/31 (H1N1) were obtained from the American Type Culture Collection (ATCC, Manassas, VA, USA). Viruses were passaged a maximum of two times in eggs. Allantoic fluid was clarified by low-speed centrifugation, aliquoted and stored at −80 °C until used. Virosomes derived from H5N1 (A/Vietnam/1194/2004) were kindly obtained by Crucell Switzerland AG, (Berne, Switzerland). Hemagglutination assays were performed using freshly isolated chicken erythrocytes at a final concentration of 1% and U-bottom shaped 96-well plates (Greiner, Nürtingen, Germany). Tissue culture infection dose_50_ (TCID_50_) were determined with Madin-Darby canine kidney cells (MDCK, ATCC) in 96-well plates (5 replicates) in the presence of bovine serum albumin (BSA, 0.125%, Intergen, NY, USA), 1mM HEPES (Invitrogen, Basel Switzerland) and 1 μg/mL L-1-tosylamide-2-phenylethyl chloromethyl ketone trypsin (Sigma-Aldrich, Buchs, Switzerland). 72 h p.i., the cells were washed and stained with crystal violet. The TCID_50_ titers were calculated according to the Reed-Muench formula (WHO Manual). All influenza viruses used in this study were handled under BSL-3 conditions. MDCK cells were maintained in minimal essential medium (MEM, Invitrogen) supplemented with 10% fetal bovine serum (FBS, Biowest, Nuaillé, France), non-essential amino-acid, 1mM sodium pyruvate and penicillin-streptomycin (all from Invitrogen). Human embryonic kidney cells 293T (ATCC) were propagated in Dulbecco’s MEM (Invitrogen) supplemented with 10% FBS.

### 3.2. Virus Inactivation

H5N1 TT05 virus was chemically inactivated with 2-bromoethylamine hydrobromide (BEI, Fluka, Buchs, Switzerland). Briefly, 1 M BEI solution was freshly prepared in 0.175 M Sodium hydroxide at pH 9 and incubated for 1 h at 37 °C to ensure proper circularization. BEI was used at a final concentration of 3 mM and incubated for 24 h at room temperature (RT) in a fume hood. The reaction was stopped by addition of 10% (volume/volume) of 1 M Na_2_S_2_O_3_ for 1 h at RT [14–16]. Inactivation was controlled on MDCK cells by absence of cytopathogenic effect after incubation for 72 h. UV inactivation was performed by irradiating virus 5 cm under a UV-lamp on ice for 11 min (10 mJ). Heat-inactivation was performed in a water-bath at 65 °C for 1 h. All inactivated viruses were assessed for infectivity and hemagglutination after treatment.

### 3.3. Reverse Genetic Viruses

All viruses generated by reverse genetics are listed in Table 2. The SA specific virus pair R1 and R2 is composed of the HA and NA from A/Hong Kong/68 (H3N2) and the remaining viral genes (or segments) from A/WSN/33 (H1N1). The R1 has wild type sequence and α-2,6-SA binding specificity, while the HA of R2 has an L226Q and S228G amino acid change in the HA leading to α-2,3-SA binding specificity [11]. The LPAI H5N1 A/duck/Hokkaido/Vac-1/04 (H5N1-Vac), composed of the HA, NP, M, PA, PB1 and PB2 from A/duck/Mongolia/54/01 (H5N2), and NA and NS from A/duck/Mongolia/47/01 (H7N1) [12] and the HPAIV A/chicken/Yamaguchi/7/04 (H5N1) [13] were kindly obtained from Dr. Y. Sakoda (Hokkaido University Japan) as two complete sets of 8 plasmids. Using reverse genetics, based on the plasmid system established by Hoffman *et al.* [17], with permission of Dr. R. Webster (St. Jude Children’s Research Hospital, Memphis, TN), a number of new viruses listed in Table 2 were generated as described [18]. Briefly, 293T and MDCK cells were mixed (1:1) in MDCK medium and transfected with 1 μg of plasmid DNA using Lipofectamine 2000 (Invitrogen) or Fugene HD (Roche, Switzerland) following manufacturer’s instructions. 24 h after transfection, 1 ml of OptiMem (Invitrogen) containing TPCK-trypsin was added to the cells (final concentration 2 μg/mL). Reverse genetic viruses were rescued after 72 h and further propagated in eggs.

### 3.4. Viral RNA Quantification

Viral RNA of different virus stock was extracted using a commercial kit Total RNA isolation with Nucleospin RNA II (Macherey Nagel, Oensingen, Switzerland) following manufacturer’s instructions. M1 and EGFP RNA were amplified by real-time reverse transcriptase PCR using QuantiTect Probe RT-PCR MasterMix (Qiagen, Hombrechtikon, Switzerland) and primers targeting conserved region of M1 as described [19,20]. Threshold cycle (CT) values were corrected to the amount of EGFP RNA.

### 3.5. pDC Enrichment and Stimulation with Influenza

Peripheral blood mononuclear cells (PBMC) were isolated from blood of specific pathogen free pigs kept at the institute, using Ficoll-Paque (1.077 g /L, Amersham Pharmacia Biotech AG, Dubendorf, Switzerland) density centrifugation and pDC were enriched 10-fold to 2–5% by CD172a sorting using magnetic antibody cell sorting (MACS) and LD depletion columns (Miltenyi Biotec, Germany) as previously described [21]. Enriched pDC were plated at 2.5 × 10^5^ cells/well in a 96-well plate, at 1 × 10^6^ cells/well in a 12-well plate or at 2 × 10^6^ cells/well in 6-well plates in MDCK medium complemented with HEPES, β-mercaptoethanol and BSA. The cells were stimulated with various hemagglutination unit doses (HAU) of the different IAV. In some experiments, heat-inactivated FBS (10%, Sigma Chemicals, Buchs, Switzerland) was added 1 h post stimulation. In the experiments with NA, *Vibrio cholerae* NA (N6514, Sigma-Aldrich, USA; concentration ranging from 2 mU to 200 mU) was added to the cells and incubated for 1 h to remove SA residues of pDC prior to virus addition. Cell supernatants were harvested 24 h post stimulation, and an IFN-α enzyme-linked immunosorbent assay (ELISA) was performed as previously described [21]. CpG D32 (10 μg/mL, Biosource Int., Camarillo, CA) was added as a positive control for pDC activation.

### 3.6. Flow Cytometry

pDC were identified as CD172a^low^CD4^high^ cells, as described by Summerfield *et al.* [22]. The following monoclonal antibodies (mAb) were used anti-CD172a (SWC3-IgG1) (clone 74-22-15, ATCC), anti-CD4 (clone 74-12-4, ATCC and clone PT90A, VMRD, Pullmann WA, USA) and anti-nucleoprotein (NP; clone HB65, ATCC). Antibody reactions were detected using isotype-specific anti-mouse F(ab)2 antibodies conjugated to isothiocyanate (FITC), R-phycoerythrin (RPE) or biotin (all from Southern Biotech; Birmingham, AL, USA) as described [23]. Biotin was detected using streptavidine-PE-Cy5 (Southern Biotech). For the NP detection, the cells were fixed and permeabilized using the Fix & Perm cell permeabilization kit (AN DER GRUB Bio Research, Vienna, Austria) according to the manufacturer’s instructions. All data were acquired with a FACSCalibur (Becton Dickinson, Basel, Switzerland) and analyzed with FlowJo software (Treestar Inc., Ashland, OR).

### 3.7. Statistical analyses

Box plots analysis and graphics displaying data as the median and 25th and 75th percentiles were performed with GraphPadPrism5 [9] and using one way repeated analysis of variance (ANOVA) and Bonferroni’s multiple comparison test.

## 4. Discussion and Conclusions

Among cells of the innate immune system pDC possess unique capacities in terms of virus sensing and IFN type I production making this cell type an important player against many viruses including IAV. Here, we demonstrated that porcine pDC, a natural host of IAV, efficiently sense and respond to various types of IAV originating from avian and mammalian sources, and that the virus dose greatly influences the magnitude of IFN-α response. Surprisingly, when plotted against HAU, all AIV tested behaved similarly in their induction of IFN-α response despite different pathogenicities and original hosts. Interestingly, at low doses we observed a statistically relevant higher IFN-α response to avian isolates, especially with H5N1, when compared to viruses isolated from swine and man. This would relate to the works published for human pDC [24,25], at least for the low virus doses. To our knowledge, the virus-dose dependency has not been documented previously. Considering the efficient sensing of AIV by pDC at low doses, one could speculate that innate immune responses would occur earlier during an infection thus helping to protect mammals against the vast majority of AIV. However in contrast with other AIV, the particular tropism of the HPAIV H5N1 for mammalian hosts and cells may relate to the observed infection of humans associated with high mortality. Our group has previously shown an H5N1 tropism for human endothelial cells [18], which would together with its potential to disseminate to other organs, enhance the probability of encountering pDC in the lung, the blood and in the lymph. In addition, the relatively higher tropism of H5N1 for macrophages and DC needs to be considered [26,27].

Theoretically, the more efficient sensing of AIV by pDC could result in a more efficient early anti-viral state induction and improve the quality of the immune responses and consequently prevent transmission. Alternatively, when avian influenza H5N1 viruses have access to the deeper areas in the lung as well as lymphoid tissue they may trigger an overproduction of cytokines resulting in a dysregulation of the immune responses and tissue damage. The exact role of pDC during influenza infection remains a matter of debate (reviewed in [26]). In murine models, pDC have been shown to be the major source of type I IFN during IAV infections [7], although other studies proposed that respiratory epithelium is the main source of IFN *in vivo* [28]. They have been shown to possess the capacity to activate and expand influenza virus-specific CD4 and CD8 T cells [29], and also have been shown to be able to present viral antigens to CD8 T cells [30]. However, mouse model questioned their protective role in immunity against influenza virus [28,31]. On the other hand, with respect to their role in pathogenesis, recent work demonstrated their potential involvement in enhanced mortality during lethal infection by limiting T cell response through a Fas-dependent apoptotic pathway, at least at a certain virus dose [32].

It is nevertheless important to note that the situation might be different in natural hosts of IAV. This is because the mice used were deficient in the Mx gene, a crucial gene in type I IFN responses providing protection against IAV infection in mice [33,34]. It is known that in human and pigs a correlation of high levels of IFN-α in respiratory secretions and symptom severity indicate a possible role of IFN-α in disease pathogenesis [35,36]. Furthermore, in non-human primates a particularly strong inflammatory and IFN signature was observed after H5N1 infection [37]. In our study we used cells isolated from pigs, representing a natural host and a relevant model for human influenza. Pigs are naturally infected by IAV, for which H1N1 and H3N2 represent the two major virus lineages adapted and circulating within the swine population. Like for human H5N1 infection, transmission between pigs was inefficient illustrating a lack of adaptation of this virus to mammalian hosts. However, experimental infection of pigs indicated a resistance against H5N1 with no induction of severe disease [38,39].

Our study also demonstrates that while virus replication is not required, viral RNA is the only trigger of IFN-α production by pDC, as virosomes free of viral RNA did not induce IFN-α. This relates to the dependency of IAV induced pDC activation on TLR7 expression found in mouse models [6]. On the other hand, differences in RNA quantity detected in the different virus stocks used in this study did not explain the different levels of IFN-α observed when low doses of avian and mammalian viruses were compared.

Considering that the data of Sandbulte *et al.* [24] indicated no role of NS1 for the differences between human IAV and H5N1, we hypothesized a possible role for HA in these differences. H5 has been previously shown to increase the tropism of IAV for non-classical target cells such as endothelial cells [18], and would certainly be required to deliver RNA to the endosomal TLR7. On the virus side, we demonstrated that the functionality of the HA was crucial for the IFN-α responses. The complete abolition of pDC response by the 65 °C heat-treatment, a treatment which also destroyed the ability of the viruses to hemagglutinate, confirmed the importance of the HA structure in the delivery of RNA inside a host cell for subsequent endosomal TLR7 sensing. Moreover we identified intact SA as an important element for pDC activation, although our results also indicate the involvement of other receptors in particular for the H7N1 viruses. This may be mediated through c-type lectins expressed on DC as has been proposed by others [40,41]. With respect to SA, we did not find any clear preference towards typical avian α-2,3 and human α-2,6 SA residues using SA-specific virus pairs This was also related to strong staining of pDC by both Maackia Amurensis lectin II and Sambucus Nigra lectins (data not shown).

Our results confirm that to stimulate pDC replicating virus is not required, but particles containing viral RNA. While *in vivo* this supports the particular strength of pDC in sensing in IAV infections, this also has an implication for *in vitro* experiments. It is important to note that IAV stocks with similar infectious titers can significantly differ in their content of defective viral particles, which contain RNA but cannot replicate. With this, dosing of the virus for matters of comparison of different IAV is a problematic issue. Certainly, a measurement including defective virus particles is more appropriate than an infectivity-based dosage. Nevertheless, we are aware that HAU are also not optimal due to their relatively weak precision and due to differences in IAV in this biological property. On the other hand, also viral RNA quantified by real-time RT-PCR did not correlate well to the ability to simulate pDC. A problem with this method could be that it is unknown how much viral RNA is present that is not inside a functional viral envelop. We concluded from our results that employment of HAU was the best available method to dose IAV for pDC stimulation.

We also demonstrate that H5N1 and H7N1 viruses are more efficient at infecting pDC when compared to a human H1N1, relating to their higher sensing capacity in terms of IFN-α responses at low virus doses. Also Sandbulte *et al.*, demonstrated higher levels of M2 viral RNA in H5N1 infected pDC when compared to human viruses [24]. However, using different reverse genetic virus pairs bearing the HA of either the H5 derived from an LP or an HPAIV, or the HA derived from a human H3 virus we did not identify a clear role for the HA in IFN-α response by pDC. Thus, future studies are required to address alternative viral genes of importance such as the NA or the polymerase genes.

Altogether, the present work illustrates the peculiar biology of the interaction of IAV with different host tropism with pDC and will help to understand the complexity of virus-host relationship for this zoonotic virus infection.

## Figures and Tables

**Table 1.** List of influenza A virus (IAV) isolates used in this study.

**Host**	**Subtype**	**Complete Name**	**Designation^a^**	**HAU**	**TCID_50_/mL**	**CT Values^b^**
avian	HP H5N1	A/turkey/Turkey/05	H5N1 TT05	1280	7.9 × 10^6^	12.51 ± 0.05
	HP H5N1	A/cygnus/Italy/742/06	H5N1 CI06	1280	5.0 × 10^7^	11.76 ± 0.04
	HP H5N1	A/mallard/Italy/835/06	H5N1 MI06	640	3.2 × 10^4^	13.11 ± 0.02
	HP H7N1	A/turkey/Italy/4580/98	H7N1 TI98	1280	3.2 × 10^6^	14.24 ± 0.09
	HP H7N1	A/ostrich/Italy/2332/00	H7N1 OI00	1280	2.0 × 10^7^	13.17 ± 0.10
	LP H7N1	A/turkey/Italy/3675/99	H7N1 TI99	320	3.2 × 10^4^	14.51 ± 0.02

human	H1N1	A/New Caledonia/20/99	H1N1 NC99	320	3.2 × 10^6^	18.28 ± 0.18
	H3N2	A/Wisconsin/67/05	H3N2 WS05	1280	3.2 × 10^6^	12.86 ± 0.06
	H3N2	A/Hong Kong/8/68	H3N2 HK68	320	1.3 × 10^6^	17.17 ± 0.15

porcine	H1N1	A/swine/Iowa/1976/31	H1N1 SI76	640	5.0 × 10^6^	16.54 ± 0.06
	H1N1	A/swine/Belgium/1/98	H1N1 SB98	320	5.0 × 10^6^	16.32 ± 0.06
	H3N2	A/swine/Flanders/1/98	H3N2 SF98	640	3.2 × 10^6^	17.50 ± 0.05

a subtype, first letters for the host and country, and year of isolation;

b determined from 100 μL of virus stock by qRT-PCR using M2 primers, ± standard deviations of triplicates.

**Table 2.** List of reverse genetic viruses used in this study.

**Designation**	**Subtype**	**Segment Substitution**	**Mutations**	**SA Binding Properties**	**Backbone**	**Cleavage Site**
R1	H3N2	H3/N2 from HK68	None	α-2,6	H1N1 WS33	Monobasic
R2	H3N2	H3/N2 from HK68	L226Q/S228G^d^	α-2,3	H1N1 WS33	Monobasic
Vac	H5N1	N1/NS from H7N1^a^	None	α-2,3	H5N2^b^	Monobasic
HAYam_Vac	H5N1	H5 from H5N1^c^	None	α-2,3	Vac	Polybasic
H3R1_Vac	H3N1	H3 (R1) from HK68	None	α-2,6	Vac	Monobasic
H3R2_Vac	H3N1	H3 (R2) from HK68	L226Q/S228G^d^	α-2,3	Vac	Monobasic

a A/Duck/Mongolia/47/01 [12];

b A/Duck/Mongolia/54/01 [12];

c A/Chicken/Yamaguchi/7/04 [13];

d Tropism for α-2,3 linked SA [11].
